# A Comprehensive Review on Bioactive Compounds Found in *Caesalpinia sappan*

**DOI:** 10.3390/molecules28176247

**Published:** 2023-08-25

**Authors:** Twinkle Vij, Pawase Prashant Anil, Rafeeya Shams, Kshirod Kumar Dash, Rhythm Kalsi, Vinay Kumar Pandey, Endre Harsányi, Béla Kovács, Ayaz Mukarram Shaikh

**Affiliations:** 1 Department of Food Technology and Nutrition, Lovely Professional University, Phagwara 144411, Punjab, India; 2 MIT School of Food Technology, MIT ADT University, Pune 412201, Maharashtra, India; 3 Department of Food Processing Technology, Ghani Khan Choudhury Institute of Engineering and Technology (GKCIET), Malda 732141, West Bengal, India; 4 Division of Research & Innovation (DRI), School of Applied & Life Sciences, Uttaranchal University, Dehradun 248007, Uttarakhand, India; 5 Department of Bioengineering, Integral University, Lucknow 226026, Uttar Pradesh, India; 6 Agricultural Research Institutes and Academic Farming (AKIT), Faculty of Agriculture, Food Science and Environmental Management, University of Debrecen, 4032 Debrecen, Hungary; 7 Faculty of Agriculture, Food Science and Environmental Management, Institute of Food Science, University of Debrecen, 4032 Debrecen, Hungary

**Keywords:** sappan wood, Brazilwood, Suou, Indian Redwood, heartwood, chemical constituents, coloring agent

## Abstract

Sappan wood (*Caesalpinia sappan*) is a tropical hardwood tree found in Southeast Asia. Sappan wood contains a water-soluble compound, which imparts a red color named brazilin. Sappan wood is utilized to produce dye for fabric and coloring agents for food and beverages, such as wine and meat. As a valuable medicinal plant, the tree is also known for its antioxidant, anti-inflammatory, and anticancer properties. It has been observed that sappan wood contains various bioactive compounds, including brazilin, brazilein, sappan chalcone, and protosappanin A. It has also been discovered that these substances have various health advantages; they lower inflammation, enhance blood circulation, and are anti-oxidative in nature. Sappan wood has been used as a medicine to address a range of illnesses, such as gastrointestinal problems, respiratory infections, and skin conditions. Studies have also suggested that sappan wood may have anticarcinogenic potential as it possesses cytotoxic activity against cancer cells. Based on this, the present review emphasized the different medicinal properties, the role of phytochemicals, their health benefits, and several food and nonfood applications of sappan wood. Overall, sappan wood has demonstrated promising medicinal properties and is an important resource in traditional medicine. The present review has explored the potential role of sappan wood as an essential source of bioactive compounds for drug development.

## 1. Introduction

Sappan wood is typically found in Southeast Asia and the Pacific Islands. The scientific name of sappan wood is *Caesalpinia sappan* L. (genus Fabaceae), and it is also known as Sappan, Brazilwood, and Suou in diverse parts of the world. Southeast Asia, Malaysia, India, and Indonesia are home to the native variety of the tropical heartwood sappan tree, also known as the Indian Redwood [1,2]. Due to its commercial and cultural significance, sappan wood has been extensively harvested in many parts of Southeast Asia, leading to concerns about its sustainability. The tree is harvested every 6–8 years and when the trunk has attained 5–6 cm diameter. The tree is cut about 1 m above the ground to allow sprouts to grow from the stump. The main branches along with the stump are harvested. The average yield of inner pulp is about 80 kg/tree. Seeds can be harvested right from the second year of planting, but the heartwood is ready only after 6–12 years. A yield of 2000–2500 kg of pods may be obtained, which in turn may yield 200–250 kg of seeds per hectare. The harvested wood is chipped into pieces, and the dye is extracted by boiling them in water. While extracting, a few paddy grains are thrown into the boiling liquid to check the completion of the extraction process. If the husk scales are off, boiling is considered sufficient. The wood dye yield varies with varietal and cultural factors. To assure the long-term survival of this tree, campaigns are being launched to support ethical harvesting methods and protect its natural habitat [2]. This plant thrives at an elevation of 1000 m above sea level and is well suited for mountainous regions with moderate temperatures. It is a small tree, typically reaching a height of 5–10 m with a diameter of 15–25 cm. The trunk and branches are spiny and covered in reddish-brown hairs, while the stem is round and brownish-green in color. The flowers are arranged in panicles, which are terminal (at the ends of branches) and located in the axils of the upper leaves. The panicles measure about 30–40 cm in length, and the pedicles (flower stalks) are approximately 1.3–1.5 cm long. The stamens (male reproductive parts) are delicate, waxy-white, and have filaments that are densely woolly at the base. The pods, measuring 7–10 cm in length and 3.8–5 cm in width, are woody, slanted, and elongated [3].

### Bottom of Form

Sappan wood has been used for various purposes for centuries, including as a natural dye, medicinal herb, and in traditional medicine practices [4,5]. The heartwood contains a natural dye called brazilin, which produces a range of red hues. Sappan wood has also been used for dyeing textiles made from using silk and cotton, producing high-quality furniture and decorative items, and coloring foods, like rice and noodles, in Southeast Asia. Additionally, the dye is employed in the food business to tend items like cheese, smoked fish, and meat [6,7]. Sappan wood has been used in traditional medicine practices for its anti-inflammatory, antioxidant, and antimicrobial properties. Many diseases, including cough, fever, gastrointestinal issues, and skin conditions, have all been treated with it [8]. Additionally, it has been employed as a blood cleanser and astringent [3,9].

The United States Department of Agriculture states that the *Caesalpinia sappan* L. classification is as follows: Kingdom (Plantae), Subkingdom (Tracheobionta), Division (Magnoliophyta), Class (Magnoliopsida), Order (Fabales), Family (Fabaceae—pea family), Genus (*Caesalpinia* L.), Species (*Caesalpinia sappan* L.—Sappan wood) [10]. *Caesalpinia sappan* L. has different vernacular names in different regions varying by language, including English (sappan wood), Tamil (Patungam), Hindi (Bakam), Telugu (Vakama), Malayalam (Sappanam, Pathimukham), Sanskrit (Patrangah, Patangah), Kannada (Chappanga), and Gujarati (Patang) [11]. The sappan wood plant is a small tree with a spherical and brownish-green stem, pubescent rufous limbs, and a height of 5 to 10 m. This wood is excellent for turning and also yields a crimson dye. It is hard, weighty, prickly, hefty, and orange-red. After a year of growth, fruit production can begin, typically during the rainy season. Flowering can then occur after about six months. Due to its cultural and economic significance, sappan wood has been designated as a state tree in some Indian states, including Kerala and Tamil Nadu [11]. Due to overharvesting and habitat loss, sappan wood is regarded as a threatened species in many areas of Southeast Asia. Efforts are underway to promote sustainable harvesting practices and conserve this tree’s natural habitats. Some countries have also established protected areas to preserve sappan wood populations. Using sappan wood for dyeing fabric has a long history in Southeast Asia. In some countries, such as Thailand, the traditional dyeing process using sappan wood is still practiced today [12]. The purpose of this review is to discuss the various bioactive components found in sappan wood, as well as the therapeutic properties of sappan wood. The conventional and novel extraction techniques of sappan wood’s bioactive components are addressed. Furthermore, the commercial uses of sappan wood are highlighted.

The conservatively accepted *Caesalpinia sappan* L. has medicinal properties based on the literature basis. Various studies have proved the uncountable benefits of sappan wood, but the exact mechanism behind the therapeutic benefits is still unknown. The use of sappan wood has begun in the cosmetic industry, but its impact should not be limited to it. It has potential to flourish in the pharmaceutical and nutraceutical industries as well. Future research will be required to determine the mechanism of action and isolation of active ingredients from *Caesalpinia sappan* L., which has extraordinarily stimulated biological effects and a significant body of traditional myths based on natural resources [13].

## 2. Bioactive Compounds of Sappan Wood

### 2.1. Bioactive Compounds of Sappan Wood

**Figure 1:**
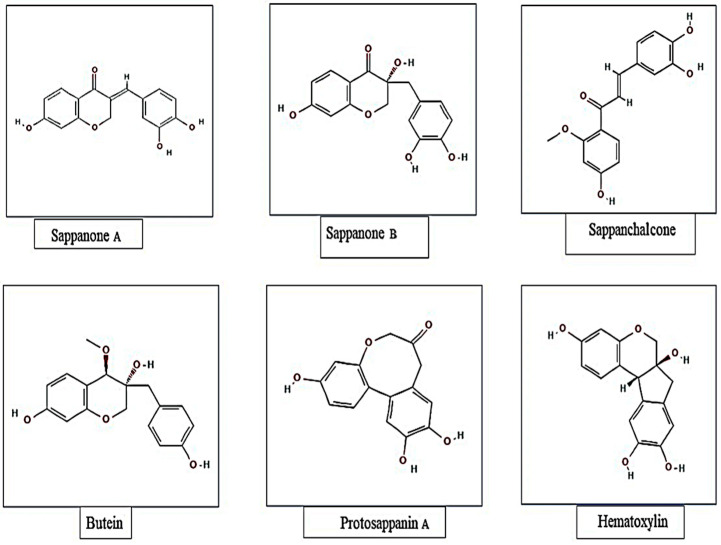
Major metabolites recognized from *Caesalpinia sappan* with identified chemical structures.

The phytochemical composition of sappan wood has been studied extensively, and it has been found that it contains various bioactive compounds. The major constituents of sappan wood are flavonoids, phenolic acids, and anthraquinones. The major metabolites recognized from *Caesalpinia sappan* L. with identified chemical structures are presented in Figure 1.

Other substances found in sappan wood, in addition to flavonoids and phenolic ones, are triterpenoids, steroids, alkaloids, saponins, and tannins. Many different biological actions, such as anti-inflammation, anticancer, and antioxidant characteristics, are exhibited by the class of polyphenolic compounds and have been connected to several health benefits [14]. Several structurally unique phenolic components, such as brazilin, xanthone, one coumarin, chalcones, flavones, and homo isoflavonoids, are present in sappan wood [15,16]. The different bioactive compounds present in sappan wood are illustrated in Table 1.

#### 2.1.1. Flavonoids

The flavonoids found in sappan wood are brazilin, haematoxylin, and protosappanin. Sappan wood includes conjugated aromatic benzene groups as mentioned above; flavonoid compounds are hypothesized to be able to block UV (ultraviolet) rays, protecting skin from exposure to the sun. The flavonoids in sappan wood have the potential to be used as sunscreens [20]. The content of flavonoid and anthocyanin components in sappan wood extract (*Caesalpinia sappan* L.) was studied. The five concentration levels used in this experiment were 20%, 40%, 60%, 80%, and 100%. Fifteen experimental units were produced after three iterations of the study. The information was shown using tables, illustrations, and descriptive statistics. The analysis revealed that the sappan wood extract had a flavonoid content of 6.02% and an anthocyanin content of 2.43% [21].

#### 2.1.2. Phenolic Acids

Phenolic acids are found in a variety of plant species and have been connected to several health benefits, including antioxidant and anti-inflammatory properties. Sappan wood contains numerous phenolic acids, including chlorogenic acid, caffeic acid, and gallic acid [22]. During research, sappan wood extract from Bone Regency, South Sulawesi Province, was extracted using ultrasonic-assisted solvent extraction (ultrasonic extraction), with water serving as the solvent. The content of polyphenols in this extract was then determined. The Follin–Ciocalteu visible spectrophotometer was employed, with pH conditions of 6, 7, and 8. The results show that the polyphenol content of sappan wood at three pH values was 34.33% (pH 6), 13.70% (pH 7), and 12.66% (pH 8). The analysis findings show that pH 6 has the highest polyphenol level and that as pH increases, polyphenol content decreases [23].

#### 2.1.3. Anthraquinones

Various pharmacological actions, such as antimicrobial, anticancer, and anti-inflammatory properties, have been linked to a family of organic compounds known as anthraquinones. Sappan wood contains several anthraquinones, including brazilin, brazilein, and sappanone A [24]. Anthroquinones, also known as simple anthrones or bianthrones, are chemical compounds. The free anthraquinone aglycones do not have much of a therapeutic effect. The sugar residue makes it easier for the aglycone to be absorbed and transported to the region of action. Anthraquinones and associated glycosides are stimulant cathartics that work by making the smooth muscle of the large intestinal wall more toned. The large intestine is where the glycosides are expelled after being reabsorbed from the small intestine, where they induce irritation of the colon mucosa and promote motility to have a laxative effect. In conclusion, rats responded to dosages of *Caesalpinia sappan* L. wood extracts by becoming laxative. All extracts except for ethanolic extract were shown to be more powerful and to only display a dose-dependent laxative effect in a drug-induced constipation paradigm. In models with low-fiber diets, all extracts showed equivalent effectiveness [25].

#### 2.1.4. Triterpenoids and Steroids

The class of organic compounds known as terpenoids is found in plants and has been shown to have a variety of pharmacological effects, such as anticancer, anti-inflammatory, and antimicrobial characteristics. Sappan wood contains several triterpenoids, including lupeol, β-amyrin, and cycloartenol [26]. Steroids are a different family of organic compounds widely distributed in the plant kingdom and exhibit various biological activities, such as anticancer and anti-inflammatory characteristics. Sappan wood contains several steroids, including stigmasterol and β-sitosterol [27].

#### 2.1.5. Alkaloids and Tannins

Alkaloids are biological complexes that contain nitrogen and are present in a variety of plant species. They demonstrate a variety of pharmacological effects, such as analgesic, anti-inflammatory, and anticancer characteristics. Sappan wood contains several alkaloids, including sappan chalcone and sappanone B [28]. Sappan chalcone has been demonstrated to have effects on the growth of human prostate cancer cells, and sappanone B has been demonstrated to have effects on inflammation by suppressing the production of proinflammatory cytokines [27]. Tannins are polyphenolic substances that are frequently present in plants and are responsible for giving them an astringent flavor. Sappan wood contains condensed tannins, sometimes proanthocyanidins, and hydrolyzable tannins, such as ellagic acid and gallic acid. Syamsunarno et al. [29] stated that the diverse range of phytochemical compounds found in sappan wood may have an extensive range of potential well-being advantages. More research is necessary to completely comprehend the compounds’ pharmacological actions and their potential therapeutic uses. During a study, the bark of the tree was successively extracted with n-hexane, ethyl acetate, methanol, and hot water before being analyzed with GC-MS to determine its total phenolic content (TPC), total flavonoid content (TFC), and total tannin content (TTC) as well as its antioxidant activity (DPPH scavenging activity). The methanol extract of the bark had the greatest concentrations of TPC (824.1662 ± 28 mg GAE/g), TFC (185.031 ± 91 mg QE/g), and TTC (987.0730 ± 98 mg TAE/g) [30].

### 2.2. Extraction of Bioactive Compounds from Sappan L.

Due to the high cost of synthetic pharmaceuticals and the negative side effects of synthetic molecules, researchers are more interested in discovering bioactive substances from natural sources, such as drugs. As a result, plant resources are continually assessed in an effort to find bioactive molecules that can treat diseases. One of the most significant bioactive natural substances found in *Caesalpinia sappan* L. heartwood is brazilin, which has a wide range of industrial applications in the textile, cosmetics, and pharmaceutical industries [31,32]. *Caesalpinia sappan* L. bioactive metabolites obtained from various parts of the plant are presented in Table 2.

*Caesalpinia sappan* L. heartwood is a dark red color that is commonly used for furniture, musical instruments, and decorative items made from wood, which is valued for its deep red hue. In addition to its esthetic properties, the heartwood of sappan wood is also known for its medicinal properties. It has been used for many years in traditional medicine to address a wide range of illnesses, such as inflammation, digestive issues, and respiratory issues [40]. The heartwood is used as a decoction in the Namya-utai solution, which possesses antithirst and cardiotonic properties. In northern Thailand, a decoction of *Caesalpinia sappan* L. heartwood, especially in the provinces of Chiang Mai, Nan, and Lampang, uses this as an anti-inflammatory drug to treat arthritis and traumatic disease. It is mostly utilized in Thailand as a coloring agent in clothing, cosmetics, beverages, and foods [41]. Traditional Chinese medicine primarily used sappan wood as an emmenagogue, hemostatic, analgesic, anti-inflammatory, and blood flow-promoting drug for traumatic diseases. Furthermore, *Caesalpinia sappan* L. heartwood decoction is used to treat a variety of conditions, including high blood pressure, cataracts, digestion, dysmenorrhea, burning sensations, ear infections, gonorrhea, heart problems, jaundice, nervous disorders, obesity, ophthalmic illnesses, stomach aches, syphilis, spermatorrhoea, urinary diseases, and vascular diseases [42]. Heartwood contains a variety of compounds, including brazilin and brazilein, which are known to have anti-inflammatory and antioxidant properties. Overall, the heartwood of sappan wood is a valuable natural resource with both esthetic and medicinal properties. Its sustainable use is important for preserving both its cultural and ecological significance [15]. The three main water-soluble flavonoids in heartwood are brazilin, protosappanin, and hematoxylin.

#### 2.2.1. Extraction of Bioactive Compounds

##### Extraction of Anticonvulsant Compounds

Using EtOAc, n-BuOH, and water, 80% aqueous methanolic extracts from *Caesalpinia sappan* wood were fractionated. This wood exhibited exceptional anticonvulsant efficacy. One of them, succinic semialdehyde dehydrogenase (SSADH), and succinic semialdehyde reductase (SSAR), were strongly inhibited by the ethyl acetate fraction. The separation of the two major active components was accomplished using many column chromatographies for the percentage determined by an activity test. Based on spectrum data, their chemical structures were identified as sappanchalcone and brazilin. The SSAR activities were inactivated by the pure compounds sappanchalcone and brazilin in a dose-dependent manner, whereas SSADH was only partly inhibited by sappanchalcone and not by brazilin [43].

##### Brazilin

**Figure 2:**
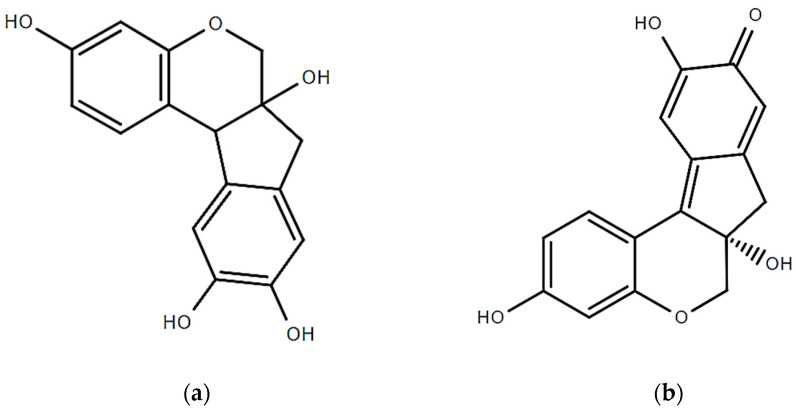
Structure of (**a**) brazilin and (**b**) brazilein.

Sappan wood (*Caesalpinia sappan* L.) contains several active compounds, but the most notable one is brazilin. The structures of brazilin and brazilein are presented in Figure 2. Brazilin is a red pigment that gives sappan wood its characteristic red color. It is a flavonoid that is part of a class of substances called chalcones. Brazilin has been found to have various biological activities, including anticancer, antioxidant properties, and anti-inflammatory ones [44]. Its medicinal advantages have also been utilized in traditional medicine. Brazilin is a white phenolic molecule with a 5-membered ring that contains two aromatic rings and one pyron. But when the hydroxyl group of brazilin is oxidized, it changes to the carbonyl group resulting in its structural change and the production of brazilein is formed (a colorful substance). Brazilein has a long history of use as a natural colorant [45]. The Caesalpinia wood-based red dye has been rendered attainable by brazilein. Currently, brazilein is regarded to be yellow. Both substances are tetracyclic because they have two aromatic rings, one pyrone ring, and five carbons. The presence of the carbonyl group causes a rise in the delocalization of electrons, transforming the yellow brazilin into the red brazilein [15]. In 95% ethanol for two hours, wood extraction yield is highest. The analysis of sappan wood’s chemical constituents led to the identification of many structural kinds of phenolic components, including brazilin, one xanthone, one coumarin, three chalcones, two flavones, and three homoisoflavonoids.

### 2.3. Medicinal Characteristics of Sappan Wood

Sappan wood exhibits various pharmacological properties. These characteristics are thought to be a result of the flavonoids and phenolic compounds found in sappan wood. According to researchers, sappan wood extract demonstrated potent antioxidant activity in vitro, as judged by its capacity to scavenge free radicals and prevent lipid for oxidation. Another study reported that sappan wood extract had a protective effect against oxidative damage in mice [46]. Sappan wood also has an antimicrobial activity against a variety of pathogenic bacteria and fungi. In one study, for instance, it was discovered that sappan wood extract had antibacterial activity against both Gram-positive and Gram-negative bacteria, such as Staphylococcus aureus and Escherichia coli. Another study found that sappan wood extract had antifungal activity against several strains of Candida [26]. Furthermore, sappan wood has been traditionally used to treat various diseases, including diabetes, cancer, and cardiovascular diseases. The studies have reported on the potential health benefits of sappan wood extracts in animal models. For instance, the study found that sappan wood extract had a hypoglycaemic effect in diabetic rats. A different study found that sappan wood extract had a cardioprotective effect in mice with an induced myocardial infarction [47]. Mode of action of various pharmacological properties of *Caesalpinia sappan* L. is presented in Table 3. Overall, the diverse range of bioactive compounds found in sappan wood and their potential health benefits suggest that it may have a role in traditional medicine and as a potential source of new drugs.

#### 2.3.1. Anti-Inflammatory Properties

**Figure 3:**
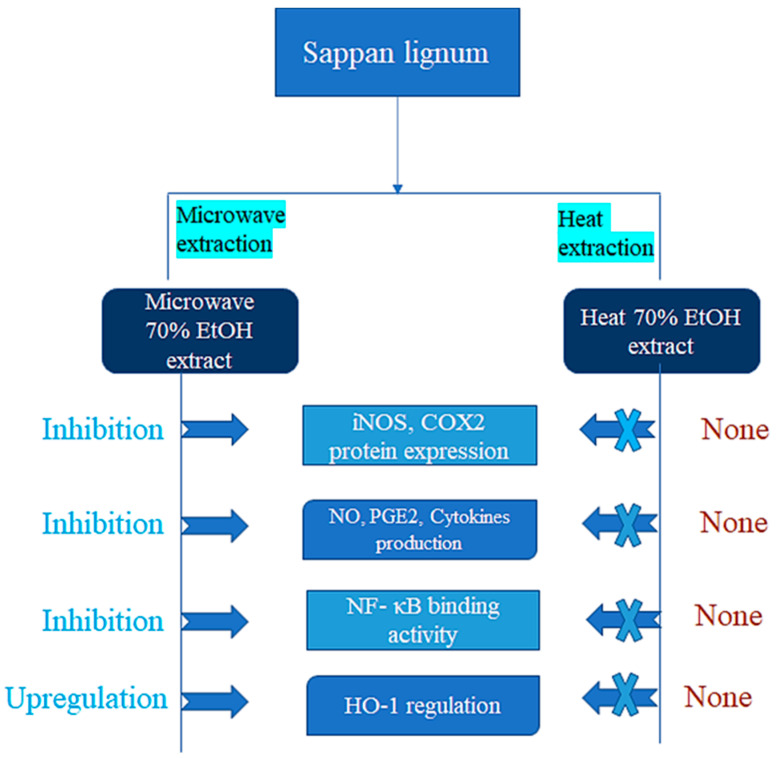
Diagrammatic representation of the extraction process that affects the *Sappan lignum* biological activity.

The heartwood of sappan wood has an extended history of use in medicine due to its anti-inflammatory properties. Additionally, sappan wood extract has been demonstrated to have analgesic (pain-relieving) properties, which can also aid in reducing discomfort brought on by inflammation [51]. Studies have shown that sappan wood contains various bioactive compounds that have anti-inflammatory effects. Brazilin is one of these compounds, and studies have shown that it can stop cells from releasing inflammatory molecules, like interleukin-1 beta (IL-1 β) and tumor necrosis factor-alpha (TNF-α). Nuclear factor kappa B (NF-κB), both crucial transcription factors that regulate the expression of genes linked to inflammation, has also been shown to be inhibited by brazilin [42]. Another component found in sappan wood called protosappanin A has been shown to stop the production of proinflammatory molecules, like cyclooxygenase-2 (COX2), nitric oxide (NO), and prostaglandin E2 (PGE2). By blocking the simulation of NF-B, protosappanin A has also been discovered to decrease the expression of proinflammatory genes, like COX-2 and inducible nitrogen oxide (iNOS) [38,50]. Moreover, sappan wood extract has been demonstrated to reduce inflammation in a variety of animal models. For instance, in a study on colitis-infected rats, sappan wood extract was shown to lessen the severity of inflammation and mucosal damage in the colon by inhibiting the activation of NF-B and the production of proinflammatory cytokines [39]. The bioactive components in sappan wood, like brazilin and protosappanin A, have been shown to inhibit the production of proinflammatory molecules and decrease the expression of proinflammatory genes by inhibiting NF-B activation [61]. These bioactive compounds are largely responsible for sappan wood’s anti-inflammatory properties [62]. The diagrammatic representation of the extraction process that affects sappan lignum biological activity is shown in Figure 3, which indicates that anti-inflammatory actions on 70% ethanol microwave extraction is more effective than 70% ethanol heat extraction.

#### 2.3.2. Antioxidant Properties

One of the properties of sappan wood is its antioxidant activity. This refers to the wood’s capacity to combat hazardous molecules known as free radicals that can hurt cells and decrease the number of diseases. Several studies have examined sappan wood’s antioxidant activity and found that it contains various compounds that can act as antioxidants. For example, sappan wood contains compounds, such as brazilin, protosappanin, and sappanone, which have antioxidant effects [51]. Sasaki et al. [49] discovered that the methanolic extract of sappan wood demonstrated strong antioxidant activity in a number of in vitro assays, including the DPPH radical scavenging assay, the ABTS radical scavenging assay, and the reducing power assay. Settharaksa et al. [46] estimated the sappan wood extract antioxidant activity by using DPPH (2,2-diphenyl-1-picrylhydrazyl) radical scavenging assay and FRAP (ferric-reducing antioxidant power) assay. The results showed that the extract possessed significant antioxidant activity in both assays.

Another study investigated the antioxidant activity of sappan wood extract. In cooked ground beef, researchers found that the extract was able to significantly reduce lipid oxidation and improve the sensory quality of the beef [62]. Another investigation evaluated the antioxidant activity of sappan wood extract in human colon cancer cells. The extract may have promise as a natural anticancer agent because the researchers discovered that it was able to cause cell death in cancer cells via an oxidative stress pathway [63]. Similarly, in human skin cells, they found that the extract could protect cells from the oxidative harm caused by UV rays, suggesting that it might be useful as a natural sunscreen ingredient [64]. Sarumathy et al. [65] also investigated the antioxidant activity of sappan wood extract in rats with liver damage caused by acetaminophen. According to their research, they found that the extract was able to significantly lessen oxidative stress and liver damage in rats. The extract may have promise as a natural treatment for liver diseases.

#### 2.3.3. Antiacne Properties

The heartwood of the tree is used to treat skin disorders, such as acne. Numerous studies have investigated the antiacne activity of sappan wood extract. Mitani et al. [54] observed that sappan wood extracts have anti-inflammatory and antibacterial effects on propionibacterium acnes, the bacteria that cause acne. According to the study, the extract has significant anti-inflammatory and antibacterial capabilities, as well as the capacity to inhibit the development of *P. acnes*. They concluded that sappan wood extract could be a promising natural substitute for the treatment of acne. Madhubala et al. [55] discovered that the compounds had powerful antibacterial and anti-inflammatory properties, suggesting that they could be used as acne treatment options. Several compounds were isolated and tested for their antiacne activity from sappan wood extract. Pattananandecha et al. [66] discovered that the extract significantly reduced inflammation and antibacterial effects on microorganisms that cause acne by using sappan wood extract. It has been discovered that the extract greatly decreased the production of proinflammatory cytokines and the severity of acne [52]. It has been suggested that the sappan wood extract significantly reduced the sebum quantity produced by human sebocytes [53]. Overall, these studies suggest that sappan wood extract has the potential as a natural treatment for acne due to its anti-inflammatory and antibacterial properties. To ascertain its efficiency and safety in long-term use, more studies are required.

#### 2.3.4. Antibacterial Properties

Srinivasan et al. [56] found that sappan wood extract has significant antibacterial activity against both Gram-positive and Gram-negative bacteria. The extract was found to inhibit the growth of various bacterial strains, including Salmonella typhimurium. *E. coli* and *S. aureus.* They suggested that the presence of different phytochemicals, such as flavonoids and tannins, in sappan wood extract serve as the main reason for its antibacterial action. Rina et al. [57] examined that sappan wood extract suppresses the antibacterial activity against Helicobacter pylori, a bacterium that can cause stomach cancer and peptic ulcers. The extract of sappan wood exhibits antibacterial activity against H. pylori, which was dose-dependent. The researchers suggested that the antibacterial activity of sappan wood extract may be due to its ability to inhibit the activity of enzymes involved in bacterial cell wall synthesis.

Puttipan et al. [58] examined the antibacterial action of sappan wood extract against dental caries bacteria. According to the investigators, the sappan wood extract inhibits a number of oral microbes, such as Lactobacillus Acidophilus and *S. mutans*. They claimed that the antibacterial action of sappan wood extract might be due to its ability to disrupt bacterial cell membranes and inhibit bacterial DNA synthesis. According to these studies, sappan wood extract may have broad-spectrum antibacterial activity and be successful against a variety of bacterial strains, including those that are resistant to traditional antibiotics. However, more investigation is required to establish the ideal dosage and administration strategy as well as any possible toxicity or adverse effects.

#### 2.3.5. Hepatoprotective Properties

One of its purported benefits is its hepatoprotective activity, meaning its ability to protect the liver from damage. Srilakshmi et al. [59] examined the hepatoprotective activity of sappan wood extract against liver damage induced by carbon tetrachloride (CCl_4_) in rats. The extract, as indicated by reduced liver enzyme levels and more effective histological characteristics, can significantly reduce liver damage. Researchers hypothesized that the sappan wood extract lowered lipid peroxidation and increased antioxidant enzyme levels. It may exercise its hepatoprotective effects by reducing oxidative stress in the liver. Gupta et al. [60] investigated the hepatoprotective activity of sappan wood extract against liver damage induced by paracetamol (acetaminophen) in rats. Kadir et al. [67] provide further evidence of the hepatoprotective activity of sappan wood extract and suggest that it may be a useful natural remedy for protecting the liver against various toxins and stressors.

#### 2.3.6. Other Medicinal Properties

In addition to their previous activities, from *Caesalpinia sappan* L., isolated compounds and crude extracts have been reported that exhibit antiviral, cytotoxic, anticancer, anticonvulsant, hypolipidemic, cognitive-enhancing activity, and analgesic activity [68]. The various pharmacological properties of *Caesalpinia sappan* are presented in Table 3.

### 2.4. Methods of Extraction of Bioactive Compounds of Sappan Wood

**Figure 4:**
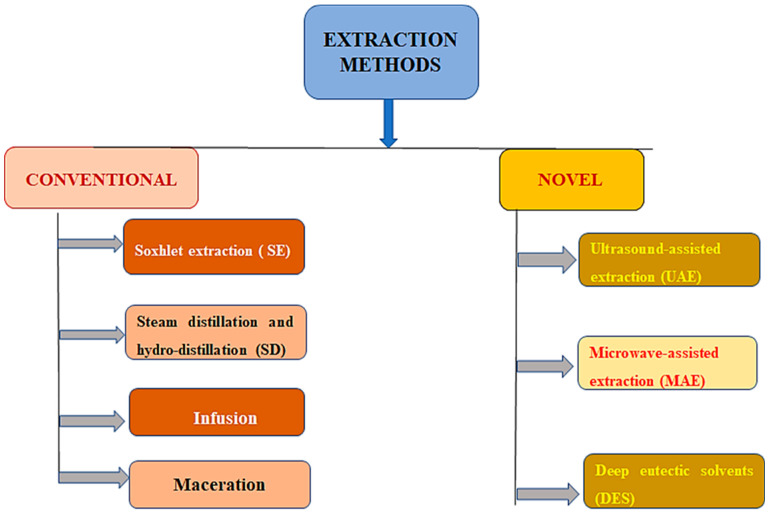
Various methods of extracting bioactive compounds.

The term “bioactive molecules” refers to substances that interact with living beings and change them in some way. The compounds can be created synthetically or obtained from natural sources, like plants and food. To discover substitutes for synthetic materials generated from natural resources, many studies have been conducted. The different procedures for the extraction of bioactive compounds from *Ceasalpinia sappan* L. are presented in Table 4. Due to the abundance of agricultural by-products that could act as a low-cost source of raw materials to extract plant bioactive compounds, this substitution may have economic advantages [69]. The antibacterial, antioxidant, and anticancer potential of bioactive substances produced by plants is well documented. Plant extracts are commonly employed in the pharmaceutical, food, and cosmetic industries [70]. The substances that are found in wood log extractives are a fusion of phenolic compounds, such as terpenoids, alkaloids, terpenes, and saponins. The extraction process is the initial step in separating and using the bioactive compounds that plants contain. In order to enhance the extraction yield of these compounds, it is crucial to use an appropriate extraction process [71]. The process yield is greatly influenced by the pretreatment techniques, the physical and chemical properties of the plant and the target complex, the choice of the extraction process, and the extraction process operating parameters [72]. Various methods of extracting bioactive compounds are presented in Figure 4.

#### 2.4.1. Conventional Extraction Technique

In this technique, procedures are found for the removal of the complexes in accordance with their varying polarities while using suitable diluters, considering that the primary component in this procedure is the solvent employed. These time-tested techniques use solid/liquid extraction techniques that have been widely used in both the laboratory and the industrial setting to isolate solid matrix chemicals. The added factors that have the most prominent effects on conventional techniques are the environmental characteristics, liquid–solid ratio, pressure, temperature, and abstraction time [71].

##### Soxhlet Extraction (SE)

To perform a Soxhlet extraction, a heat source is used to vaporize the solvent, which is then compressed in a condenser that produces reflux and discharged over the specimen’s container. Baron Von Soxhlet, who developed this method in the middle of the nineteenth century, is the source of the name for this technique [72]. The solvent containing the substance that was extracted is drawn back down to the bottom by a siphon once it reaches the top of the specimen’s container. This procedure can be carried out for a predetermined number of Soxhlet cycles or repeatedly over a predetermined number of times over a predetermined amount of time [74]. The temperature of the extraction changes depending on the solvent that is being used because the extraction is performed at the solvent’s boiling point [75]. Soxhlet extraction has a number of benefits, including the maintenance of relatively high extraction temperature due to the heat of the distillation flask, the preservation of the sample in contact with fresh solvent, the lack of filtration required after lixiviation, the effective simplicity, and the low cost. Santos et al. [75] produced 5.10 weight percent for ethyl acetate, 1.57 weight percent for n-hexane, and 7.23 weight percent for ethanol when studying the Soxhlet extraction of wood, i.e., Eremanthus erythropappus using solvents that varied in polarity. This most likely happened as the yield of Soxhlet extraction typically increases with solvent polarity, which is understandable given the poor selectivity of polar solvents. Due to this poor selectivity, there is an increase in the extraction of nonvolatile substances, like steroids, coumarins, flavonoids, tannins, saponins, and triterpenes. Similarly, Bukhanko et al. [74] studied from a biorefinery point of view the branches, cones, needles, and bark of the *Piceaabies* L. species with the goal of extracting different complexes.

##### Steam Distillation and Hydrodistillation

The most common method to extract steam from water is by boiling it all at once. The plant material, which is positioned in a separate location, is passed through by the generated vapor. Direct steam can be used as a stand-in for this process to remove the substance. But in hydrodistillation, plant materials, and water are combined in one vessel and processed simultaneously. Both of these techniques involve dissolving extracts in steam to extract the liquid [76]. Despite the techniques’ similarities, steam distillation produces more essential oils and a better mixture of volatile chemicals. Because there is no direct contact between the plant material and the water during the steam distillation process, it can prevent degradation and produce essential oils of higher quality [77]. The study of Meullemiestre et al. [69] on the hydrodistillation on Eremanthus erythropappus (DC) Macle-like wood using the particles that Tyler 28 and 32 mesh preserved (30% and 70%, respectively) attained a particle diameter of 520 m on average and weighed the sample precisely. Sawdust is used as the raw material in this process because the wood is typically pulverized before being distilled, which maximizes the extraction yield. Santos et al. [75] discovered that extraction time, which varies on an hourly basis, is another crucial element for extraction yield.

##### Infusion

A common extraction method used frequently in conventional medicines is infusion. The infusion is made by mixing a small quantity of plant matter with a solvent at a high temperature, which prepares the mixture quickly [78,79]. The plant matter must be chopped up into little bits to make the extraction of the chemicals easier. Utilizing this technique results in extracts that are rich in glycosides and essential oils [80]. The study into Prunus avium stems can provide a variety of flavonoids when heated in 200 mL of water with 1 g of the sample, then cooled for 5 min at room temperature; i.e., quercetin-3-O-rutinoside, quercetin-3-*O*-glucoside, kaempferol-3-*O*-glucoside, kaempferol-3-O-rutinoside, and catechin were among the flavonoids found [81]. Mu’nisa et al. [82] reported that sappan wood extracted using distilled solvent was conducted using infusion for 15 min at a temperature of 90 °C. Infusion was performed of sappan wood making by introducing 20 g simplicia of sappan wood into the pot infusion and adding 100 mL of distilled water into a measuring cup. The solution was heated at 90 °C for 15 min, after which it was filtered using a flannel cloth, hereinafter called the filtered water infused wooden cup. Sappan wood infusion can be stored at a temperature of 4 °C. Based on the research results, infusing sappan wood (*Caesalpinia sappan* L.) using distilled water solvent was analyzed by using a phenolic compound i.e., gallic acid standard solution of 551.663 mgGAE/mL infusion, while the analysis test flavonoid quercetin used a standard solution of 1.103 mgQAE/mL infusion. Infused sappan wood can reduce free radical DPPH. The capacity to reduce free radical DPPH sappan wood is greater than the ability of quercetin. The IC50 value of the sappan wood is 0.047 mg/mL higher than quercetin is 5054 mg/mL.

##### Maceration

It is easy to extract materials using this procedure. Although not entirely successful in removing bioactive compounds from plants, this approach has the advantage of being straightforward. The bioactive components are extracted using this approach, which involves submerging a plant sample in a solvent for a long time. In order to recover as many of the scattered compounds as possible, the liquid is drained, and the solid material that remains at the bottom is pressed [76,79]. Variable amounts of time are used during the maceration process.

Using (70:30 *v*/*v*) water–ethanol solution and maceration with n-hexane as solvents, tannins, flavonoids, and polyphenols were extracted from *Populus nigra* L. wood [83]. Bostyn et al. [84] reported that macerated sawdust could be used to create extractives, a source of various useful compounds that can be utilized in industries. Two of the most important flavonoids of *Robiniapseudoacacia* L. wood, robinetin and dihydrorobinetin, were obtained through the maceration of the wood using a water–ethanol solution as the solvent, yielding concentrations of 670 mg/L and 3000 mg/L, respectively. Extracts produced by maceration have strong antioxidant properties. Several researchers have verified that using the maceration technique, the extract yields have virtual total phenolic content (TPC) values [83,85]. Batubara et al. [86] macerated CS heartwood (500 g) powder in 5 L of MeOH for 12 h. This process was repeated twice, and the extract was concentrated by using a rotary evaporator. The crude extract (10 g) was separated on a silica gel column and eluted with hexane, ethyl acetate, and methanol. Brazilin was purified from the ethyl acetate fraction by preparative HPLC using a gradient solvent system from 5% to 100% methanol in 0.05% trifluoroacetic acid at a flow rate of 10 mL/min for 45 min.

#### 2.4.2. Novel Extraction Technique

Novel extraction methods typically operate at low temperatures. In this technique, the stability of the extracted chemicals can be preserved by reducing or eliminating the usage of organic solvents through the abstraction procedure. Furthermore, because of the high solvent temperatures and prolonged extraction times, it is possible that the bioactive components will be thermally degraded. To avoid the problems with traditional methods, different extraction techniques have been created [87]. In addition, novel techniques typically generate higher yields and higher-quality products because they use less energy and time during extraction [75].

##### Ultrasound-Assisted Extraction (UAE)

Ultrasonic wave utilization during cavitation facilitates the liberation of the required compounds by cellular rupture of plant cells through the preparation stage or during both liquid and solid extraction. This process is known as ultrasound-assisted extraction. In this process, ultrasonic waves produce cycles of extension and compress the molecules that make up the medium as they travel through the so-called cavitation phenomenon. This results in the expansion, bursting of froths, and formation in a liquid standard because of the consequence of these alternating fluctuations in pressure [88]. It has been demonstrated that ultrasound-assisted extraction is effective at obtaining a variety of products, including proteins, vital oil polysaccharides, pigments, dyes, and bioactive complexes. The benefits of utilizing UAE for liquid and solid extraction include improved yield of extraction and decreased time period, solvent intake, energy, and condensed operative temperature, which enables the abstraction of complexes that are sensitive to heat. UAE is a relatively low-cost and easy-to-use method that has the ability to use a variety of solvents. In order to evaluate the outcomes, extraction by maceration is carried out in a similar way. It was discovered that this method requires roughly 24 h to obtain yield values [89]. Kurniasari et al. [90] reported the extraction of the phenolic compound of sappan wood using ultrasound-assisted extraction (UAE). It has been considered a green technology that gives better quality products with higher extraction rates in a shorter time and with less energy. The extraction of sappan wood using a UAE probe with varying ethanol concentrations (50%, 60%, 70%, 80%, and 90% *v*/*v*) revealed that the extract yield increased with higher ethanol concentrations up to 80%. However, beyond this point, the yield decreased. When comparing the UAE and Soxhlet methods, it was observed that UAE provided a higher yield (10.33%) in a shorter duration of time (20 min) compared to the Soxhlet method, which yielded 9.67% over a longer period (180 min). Yuniati et al. [91] carried out a study using the ultrasound-assisted extraction method to optimize and characterize the process of extracting colorants from sappan wood. The research identified the optimal operating conditions for sappan wood extraction, including a frequency of 40 kHz, a temperature of 60 °C, a ratio of 0.0050 g mL^−1^, an extraction time of 20 min, and the use of a 60% ethanol solvent. The extracted sappan wood yielded a color ranging from yellow to reddish-orange under acidic pH levels (2–6), turned red under neutral pH (7), and shifted toward a purplish-red hue with increasing pH. A qualitative analysis confirmed the presence of quinone, flavonoid, quinone, and tannin compounds, as well as several phenolic compounds detected through GCMS.

##### Microwave-Assisted Extraction (MAE)

The basic devices of ionic polarization and molecular redirection are the foundation of microwave-assisted extraction. The polar molecules start to vibrate when exposed to an electrical pitch produced by microwave radiation, keeping their dipoles unceasingly allied with the electric field [92]. Microwaves are electromagnetic radiation with frequencies ranging from 0.3 to 300 GHz, with 2.45 GHz being the typical frequency utilized in commercial systems [77,93]. Moreira et al. [94] utilized water and ethanol as the solvent for the extraction of phenols from the Malus domestica Borkh heartwood using MAE with an output of 23 mg of GAE/g of dry wood extracted. According to Meullemiestre et al. [69], a 43 min MAE extraction produced an output of 0.43 percent (*w*/*w*) and 74.62 mg of GAE/g obtained in the TPC. However, after an 8 h hydrodistillation, the yield was just 0.28 percent (*w*/*w*), and the GAE TPC was 54.14 mg/g extract. So, using MAE produced an extraction yield that was comparable to or greater than hydrodistillation in a shorter time. Ahmad et al. [95] reported brazilin levels from sappan wood (*Caesalpinia sappan* L.) using the optimized ionic liquid-based microwave-assisted extraction (IL-MAE) method. According to his results, the IL-MAE (ionic liquid-based microwave-assisted extraction) method condition’s optimization was carried out using response surface methodology with the Box–Behnken design. Brazilin levels were determined by the high-performance liquid chromatography (HPLC) gradient method (0.3% acetic acid in water and acetonitrile). HPLC analysis of sappan extract showed brazilin levels of 807.56–948.12 mg/g extract. In his present study, imidazolium basic IL-MAE was optimized and first applied to elevate the brazilin levels from sappan wood. Thus, this is an optimum extraction condition for elevating sappan wood’s brazilin levels rapidly, efficiently, quickly, and in an environmentally friendly manner.

##### Extraction by Using Deep Eutectic Solvents (DES)

In order to maximize extraction efficiency, DES compositions can vary depending on the characteristics of the matrix of plants from which they will be isolated. DES is made up of nonionic types (molecular components or salts) that are connected via hydrogen bonds. This effectiveness is related to the chemical and physical characteristics of DES, including hydrogen bonding, miscibility, viscosity, density, and polarity [77]. DES is composed of multiple solid components that combine to generate a eutectic combination substance with a melting point less than the combined melting points of a separate component [96]. It has been discovered that natural-based ingredients are used to make naturally deep eutectic solvents (NADESs), improving the solvents’ environmental safety. As a result, NADES is regarded as the primary green solvent [22]. The benefits of DES include biodegradability, low cost, low volatility, ease of use, low toxicity, high stability, sustainability, recyclability, and very little vapor pressure [73]. DES based on glycerol and choline chloride was used to extract the brazilin from the heartwood of *Caesalpinia sappan* L., and ultrasound was used to monitor the process. Brazilin was produced at a rate of 368.67 g/mL, 6.4 times more than the obtained value when it was macerated with water and ethanol (57.38 g/mL), despite solvent ingestion and the duration being higher [22]. Similar methods were used in the investigation to extract brazilin from sappan wood using betain:lactic acid. They utilized betain:lactic acid for 30 min, yielding 4.49 mg/g of brazilin. They carried out a SE via water–ethanol used for 3 h, yielding 5.43 mg/g, and extraction by maceration using water–ethanol used for 72 h, yielding 4.58 mg/g. Due to the shorter extraction time and competitive extraction yield achieved, DES extraction has thus far shown to be quite appealing [73].

### 2.5. Commercial Applications

#### 2.5.1. Natural Colorant

Sappan wood is an excellent source of natural red dye. The wood contains a pigment called brazilin, which is used to dye fabrics, leather, papers, and other materials. The dye obtained from sappan wood is ecofriendly and has excellent color fastness properties, which is a glycoside hematoxylin [32,97]. Using sappan wood as a natural colorant has gained renewed interest, especially in the food industry. SWE (sappan wood extract) has been considered a safe and effective natural colorant for various food products, including confectioneries, beverages, and meat products. The utilization of SWE as a natural colorant in food products has been approved by several regulatory agencies, such as the European Union (EU) and the US Food and Drug Administration (FDA). The heartwood of the sappan tree is rich in a red pigment called brazilin, which is responsible for its vivid color. For example, in the use of SWE as a natural colorant in strawberry-flavored milk, the results depicted that SWE was effective in producing a stable red color in the milk without affecting its sensory properties. Also, SWE can be efficiently used as a natural colorant in various applications, including food, cosmetics, and textiles [7,98].

#### 2.5.2. Textile Industry

SWE has been shown to be used as a natural colorant in the textile industry. The extract can be used to dye cotton, silk, and wool fabrics, producing shades of pink, red, and purple. In addition to its coloring properties, sappan wood extract has also been found to have antibacterial and antifungal activities, making it a potential candidate for use in textile preservation [99,100].

#### 2.5.3. Beverages

SWE can be used as a natural colorant in beverages, like tea and fruit juices, without affecting their taste or aroma. The extract has also been found to have antioxidant properties, which can potentially enhance the numerous health benefits of these beverages [9,12].

#### 2.5.4. Confectionery Products

**Figure 5:**
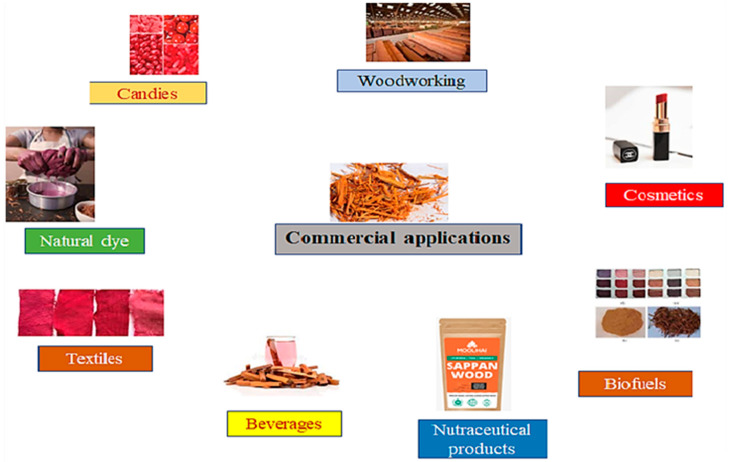
Different commercial applications of sappan wood.

SWE has been evaluated for its potential as a natural food colorant in various applications, including candies, baked goods, confectioneries, and meat products. The extract has been found to be stable under different processing conditions, and its coloring properties have been comparable to those of synthetic food colorants [101]. Thus, sappan wood is a promising natural colorant with various biological activities. Several regulatory agencies have approved its use as a natural colorant in food products, and several studies have reported its effectiveness in producing a stable red color in various food products. Different commercial applications of sappan wood are presented in Figure 5.

#### 2.5.5. Nutraceuticals

Sappan wood contains several bioactive complexes, such as terpenoids, phenolic acids, and flavonoids, with potential health benefits. Extract from sappan wood could be developed into nutraceutical products, such as dietary supplements. Sappan wood compounds have been shown to possess anti-inflammatory, antioxidant, and anticancer properties [102].

#### 2.5.6. Natural Remedies

Sappan wood is utilized in traditional medicine to treat numerous ailments, including diarrhea, inflammation, and fever. It could be developed into herbal remedies for these conditions [103].

#### 2.5.7. Biofuel

Sappan wood could be used as a source of biofuel. A study conducted in Brazil found that sappan wood has a high energy content and is suitable for use as a solid biofuel for cooking and heating [104].

#### 2.5.8. Cosmetics

Sappan wood extract has been found to possess antiaging and skin-lightening properties. It could be used as an active ingredient in cosmetic products, such as an antiaging creams, lotions, and face masks, and it might be used as an active ingredient [105].

#### 2.5.9. Woodworking

Sappan wood is a durable hardwood with a rich reddish-brown color. It is used in woodworking to make furniture, flooring, and decorative items. It is also used in traditional Japanese lacquerware, which is called Suou. The heartwood of sappan wood is dense and durable. It has high strength and stiffness and is suitable for use in structural applications [106]. Authors should discuss the results and how they can be interpreted from the perspective of previous studies and of the working hypotheses. The findings and their implications should be discussed in the broadest context possible. Future research directions may also be highlighted.

## 3. Conclusions

The conservatively accepted *Caesalpinia sappan* has medicinal properties based on the literature basis. It can be said that *Caesalpinia sappan* heartwood has significant potential for medicinal and coloring uses. It has a long history of use as a colorant in wines, meat, and textiles. In Kerala, Caesalpinia is traditionally used as herbal mineral water, which shows high therapeutic value. Medicinally, it is advised to use wood instead of logwood. It is extensively used in Ayurveda and Unani medicine. Brazilin has high latent pharmacological activities, such as antitumor, antidiabetic, anti-inflammatory, and immunostimulant properties and blood purifying action and healing properties. Various research has proved the uncountable benefits of sappan wood, but the exact mechanism behind the therapeutic benefits is still unknown. The use of sappan wood has begun in the cosmetic industry, but its impact should not be limited to it. It has a potential to flourish in the pharmaceutical and nutraceutical industries as well. Future research will be required to determine the mechanism of action and isolation of active ingredients from *Caesalpinia sappan* L., which has extraordinarily stimulated biological effects and a significant body of traditional myths based on natural resources.

## Figures and Tables

**Table 1:** Different bioactive compounds present in sappan wood.

Bioactive Compound	Properties	References
Xanthone	The xanthones existing in the pericarp, whole fruit, heartwood, and leaf of mangosteen have been found to possess a broad variety of pharmacologic properties, including antioxidant, antitumor, antiallergic, anti-inflammatory, antibacterial, antifungal, and antiviral activities (Garcinia mangostana Linn., GML).	[15]
Coumarin	Coumarin is a substance that smells like vanilla and is present in many plants. It was historically used to flavor meals.	[14]
Chalcones	The golden crystalline ketone C_6_H_5_CHCHCOC_6_H_5_, as well as many of its many derivatives, some of which are flavone-related plant colors, are created by combining benzaldehyde and acetophenone. Currently, a wide range of chalcones are used as dietary additives, cosmetic ingredients, and for the treatment of gastritis, stomach cancer, viral illnesses, cardiovascular diseases, pain, and for the treatment of pain.	[16]
Flavones	A colorless, crystalline compound that serves as the building block for several yellow or whitish plant pigments. The anti-inflammatory properties of phytonutrients, like flavonoids, are advantageous, and they shield our cells from oxidative harm that can cause diseases.	[17]
Homo isoflavonoids	Within a select few plant families, the rare compound is spread. These natural compounds can be found in large quantities in the genus Caesalpinia. Numerous bioactivities of homoisoflavonoids have been noted, including antimicrobial, antimutagenic, antidiabetic, and vasorelaxant properties.	[18]
Brazilin	C_16_H_14_O_5_, a white or pale phenolic substance, is primarily used for dyeing and is derived from the Caesalpinia Brazilwood species.	[19]

**Table 2:** *Caesalpinia Sappan* L. bioactive metabolites from various parts of plant.

Part	Principal Compound	Reference
Stem	Flavonoids, tannins, alkaloids, sterols, and terpenoids	[20]
Seeds	Caesalpinia R and S Caesalsappanins A–L Caesalsappanins M–N	[33,34,35]
Bark	Alkaloids, flavonoids, tannins, terpenoids, and steroids	[27]
Wood	Brazilin, sappanone B, and protosappanin A	[36]
Leaves	Glycosides, phenols, tannins, saponins, flavonoids, and steroids	[11]
Heartwood	Ceasalpiniaphenols A–D Sappanchalcone, ceasalpiniaphenol G, and quercetin Brazilin, protosappanin A, protosappanin B, protosappanin C, protosappanin D, and protosappanin E 3′-Deoxy-4-O-methylepisappanol, (+)-(8S,8′S)-bisdihydrosiringenin Brazilein palmitic acid Protosappanins E-1 and E-2 Lupeol, vanillin, β-sitosterol, linoleic acid, stigmasterol, and friedelin	[37,38,39]

**Table 3:** Mode of action of various pharmacological properties of *Caesalpinia sappan* L.

Characteristics	Mode of Action	References
Anti-inflammatory	Inhibition of iNOS gene expression Nuclear factor kappa B (NF-κB) Tumor necrosis factor-α production	[38,42,48,49,50] [38,42,50,51]
Antioxidant	DPPH radical scavenging assay Ferric reduction assay	[49] [46,51]
Antiacne	Zone of inhibition	[52,53,54,55]
Antibacterial	MIC (minimum inhibitory concentration)/MIB (minimum bactericidal concentration)	[56,57,58]
Hepatoprotective	Inhibition of CCl_4_ intoxication	[59,60]

**Table 4:** Different procedures for extraction of bioactive compounds from *Ceasalpinia sappan* L.

	Conventional Extraction			
Raw Material	Technique	Experimental Data	Results	References
**Conventional extraction**		Solvent: Ethanol–water	Brazilin produces	
		(80:20)	3.12 mg/g of extract.	[73]
		SLR:1:20 (*w*:*v*) 72 h		
		Solvent: Ethanol–water	Brazilin produces	
		(96:4)	4.58 mg/g of extract.	
	Maceration	SLR:1:20 (*w*:*v*) 72 h		
			Brazilin produces	[22]
	Soxhlet extraction	Solvent: Ethanol–water	5.43 mg/g of extract.	
		(96:4)		
		SLR:1:20 (*w*:*v*) 3 h		
**Novel extraction**	Deep eutectic solvents	Betain:lactic acid	Brazilin produces	
		with 60% water	4.49 mg/g of extract.	[22,73]
		SLR: 1:20 (*w*:*v*)		

Abbreviations: SLR: Solid/liquid ratio.

## Data Availability

Data will be made available on request.
